# MRI-based machine learning model: A potential modality for predicting cognitive dysfunction in patients with type 2 diabetes mellitus

**DOI:** 10.3389/fbioe.2022.1082794

**Published:** 2022-11-22

**Authors:** Zhigao Xu, Lili Zhao, Lei Yin, Yan Liu, Ying Ren, Guoqiang Yang, Jinlong Wu, Feng Gu, Xuesong Sun, Hui Yang, Taisong Peng, Jinfeng Hu, Xiaogeng Wang, Minghao Pang, Qiong Dai, Guojiang Zhang

**Affiliations:** ^1^ Department of Radiology, Radiology-Based AI Innovation Workroom, The Third People’s Hospital of Datong, Datong, China; ^2^ Graduate School, Changzhi Medical College, Changzhi, China; ^3^ Department of Endocrinology, The Third People’s Hospital of Datong, Datong, China; ^4^ Department of Materials Science and Engineering, Henan University of Technology, Zhengzhou, China; ^5^ College of Medical Imaging, Shanxi Medical University, Taiyuan, China; ^6^ Department of Radiology, First Hospital of Shanxi Medical University, Taiyuan, China; ^7^ Medical Department, The Third People’s Hospital of Datong, Datong, China; ^8^ Department of Radiology, The Second People’s Hospital of Datong, Datong, China; ^9^ Department of Radiology, Affiliated Hospital of Datong University, Datong, China; ^10^ Department of Radiology, The People’s Hospital of Yunzhou District, Datong, China; ^11^ Huiying Medical Technology (Beijing) Co. Ltd, Beijing, China; ^12^ Department of Cardiovasology, Department of Science and Education, The Third People’s Hospital of Datong, Datong, China

**Keywords:** MRI, machine learning model, mild cognitive impairment, dementia, type 2 diabetes mellitus

## Abstract

**Background:** Type 2 diabetes mellitus (T2DM) is a crucial risk factor for cognitive impairment. Accurate assessment of patients’ cognitive function and early intervention is helpful to improve patient’s quality of life. At present, neuropsychiatric screening tests is often used to perform this task in clinical practice. However, it may have poor repeatability. Moreover, several studies revealed that machine learning (ML) models can effectively assess cognitive impairment in Alzheimer’s disease (AD) patients. We investigated whether we could develop an MRI-based ML model to evaluate the cognitive state of patients with T2DM.

**Objective:** To propose MRI-based ML models and assess their performance to predict cognitive dysfunction in patients with type 2 diabetes mellitus (T2DM).

**Methods:** Fluid Attenuated Inversion Recovery (FLAIR) of magnetic resonance images (MRI) were derived from 122 patients with T2DM. Cognitive function was assessed using the Chinese version of the Montréal Cognitive Assessment Scale-B (MoCA-B). Patients with T2DM were separated into the Dementia (DM) group (*n* = 40), MCI group (*n* = 52), and normal cognitive state (N) group (*n* = 30), according to the MoCA scores. Radiomics features were extracted from MR images with the Radcloud platform. The variance threshold, SelectKBest, and least absolute shrinkage and selection operator (LASSO) were used for the feature selection. Based on the selected features, the ML models were constructed with three classifiers, k-NearestNeighbor (KNN), Support Vector Machine (SVM), and Logistic Regression (LR), and the validation method was used to improve the effectiveness of the model. The area under the receiver operating characteristic curve (ROC) determined the appearance of the classification. The optimal classifier was determined by the principle of maximizing the Youden index.

**Results:** 1,409 features were extracted and reduced to 13 features as the optimal discriminators to build the radiomics model. In the validation set, ROC curves revealed that the LR classifier had the best predictive performance, with an area under the curve (AUC) of 0.831 in DM, 0.883 in MIC, and 0.904 in the N group, compared with the SVM and KNN classifiers.

**Conclusion:** MRI-based ML models have the potential to predict cognitive dysfunction in patients with T2DM. Compared with the SVM and KNN, the LR algorithm showed the best performance.

## 1 Introduction

Diabetes is a group of metabolic diseases characterized by hyperglycemia resulting from defects in insulin secretion, insulin action, or both. Chronic hyperglycemia of diabetes is associated with long-term damage, dysfunction, and failure of different organs, especially the eyes, kidneys, nerves, heart, and blood vessels (Roden, 2016). With the aging of the population and the change in people’s living habits, diabetes mellitus has gradually become a critical health issue worldwide owing to its high morbidity, disability, and mortality (IDF Diabetes Atlas Group, 2015; Li et al., 2020). There are two types of diabetes mellitus, type 1 diabetes mellitus (T1DM), type 2 diabetes mellitus (T2DM), gestational diabetes mellitus, and other special types of diabetes. They are distinguished based on etiology and clinical manifestation. T2DM, characterized by insulin resistance (IR) and relative insulin deficiency, is the most frequent type of diabetes mellitus, accounting for no less than 90% of all types.

The association between T2DM and cognitive impairment has been established. Numerous studies have demonstrated that T2DM can increase the risk of cognitive impairment and may even progress to dementia, such as vascular dementia and Alzheimer’s disease (AD) (Stewart and Liolitsa, 1999; Strachan et al., 2011; Moran et al., 2019; Alkethiri et al., 2021).

T2DM is associated with brain abnormalities on MRI scans, containing brain structural and functional abnormalities. Some MRI markers of cerebral small vessel disease, especially lacunar infarcts, are more common in patients with T2DM (Geijselaers et al., 2015; Lawson et al., 2020). Some previous studies have focused on the brain functional changes of T2DM patients using resting-state functional magnetic resonance imaging and perfusion-weighted imaging (Chen et al., 2021; Xia et al., 2022). Furthermore, artificial intelligence (AI) combined with conventional medical imaging may be useful for detecting cognitive dysfunction in patients with T2DM.

Radiomics is an emerging field that involves the process of extracting a large number of high-dimensional mineable features from medical images and subsequently analyzed using AI methods. Radiomics workflow involves image acquisition, region of interest (ROI) segmentation, features extraction, and statistical analysis, then a statistical model is constructed based on ML or deep learning algorithms with the selected features. According to the clinical or biological question and a piece of available prior knowledge, the model is tuned. In the field of radiology, ML and deep learning algorithms were widely used (Currie et al., 2019). Specific serviceability of ML in medical imaging includes, not only extraction of radiomics features, automated image segmentation, detection and classification of lesions, and data analysis, but also providing rapid and accurate noninvasive biomarkers for some disorder risk prediction, diagnostics, prognosis, treatment response monitoring (Libbrecht and Noble, 2015; Bi et al., 2019; Choi et al., 2020; Greener et al., 2022).

However, there is a lack of a reliable predictive model based on the ML method for the detection of cognitive dysfunction in patients with T2DM. So far it is unclear whether cognitive abnormity in patients with T2DM is related to brain texture. Therefore, we consider the correlation analysis between brain texture and cognitive impairment in people with T2DM essential.

Based on previous studies, we aimed to investigate cerebral radiomics features based on MRI and construct three machine-learning models to evaluate the cognitive state in patients with T2DM. To evaluate the usefulness of the prediction model using a ML algorithm, we constructed and compared them, and subsequently, we screened out the optimal model. The model could be useful to explain and should have a potential predictive ability for cognitive impairment in patients with T2DM.

## 2 Materials and methods

### 2.1 Study population

This study was approved by The Research Ethics Committee of The Third People’s Hospital of Datong, and all the patients included in the study were provided written informed consent for the acquisition, analysis, and publishing of the anonymized data collected. Also, the study was conducted according to the declaration of Helsinki.

We enrolled 122 patients (47 men and 75 women; mean age, 63 years ± 7.07; range, 51–86 years) diagnosed with T2DM from February 2020 to July 2021 in the Center for Endocrine and Metabolic Diseases of The Third People’s Hospital of Datong, Shanxi province, China. The diagnostic criterion of T2DM patients was either fasting plasma glucose (FPG) level ≥7.0 mmol/L or 2-h oral glucose tolerance test (OGTT) glucose level ≥11.1 mmol/L (Roden, 2016). The inclusion criteria also included: 1) Age >50 years old to minimize the adverse effects of aging on cognitive function since numerous previous studies have reported that aging is a risk factor for dementia (Finkenzeller et al., 2019; Lee et al., 2020; Kaneko et al., 2021); 2) no less than 6 years of education to ensure the literacy of all subjects. 3) no history of central nervous system dysfunction or medical diseases that considerably affect neurological function, or severe heart, kidney, or liver diseases; 4)taking drugs within 3 months, such as psychoactive and steroid drugs; alcohol or drug addiction; 5) within 3 months, no taking cognition-related drugs, such as psychoactive and steroid drugs; alcohol or drug addiction; 6) ability to perform the imaging procedure following the instruction of the doctors; and 7) Right-handedness, walking independently. The exclusion criteria included: 1) type 1 or other type diabetes; 2) contraindications for MRI examination; 3) body mass index (BMI) > 35 kg/m^2^, because obesity impairs cognition (Dye et al., 2017; Ganguli et al., 2020); and 4) The acquired images could not meet the analysis requirements.

### 2.2 Clinical, anthropometric, and laboratory data

Clinical data related to diabetes were collected from the patients’ medical records. The weight status was assessed by measuring the body mass index (BMI). The blood pressure, weight, and height of each participant were measured by the standard survey method. Standard laboratory testings were carried out to measure fasting plasma glucose (FPG), glycosylated hemoglobin (HbA1c), alanine transaminase (ALT), aspartate transaminase (AST), gamma-glutamyltransferase (GGT), blood urea nitrogen (BUN), serum creatinine (SCr), total cholesterol (TC), triglyceride (TG), high-density lipoprotein (HDL), low-density lipoprotein (LDL), urine creatinine (UCr). All enrolled individuals were required to receive the Montreal Cognitive Assessment (MoCA), Rey Auditory Verbal Learning test (RAVLT), Activities of Daily Living (ADL), and Clinical Dementia Rating (CDR) scale test independently. Furthermore, duration of diabetes, history of smoking, whether or not complicated with coronary heart disease, retinopathy, or intermittent claudication were also recorded.

### 2.3 Cognitive impairment assessment

The Montréal Cognitive Assessment (MoCA) is one of the most widely used screening tests for cognitive impairment around the world (Nasreddine et al., 2005; Zhai et al., 2016; Kopecek et al., 2017; Pinto et al., 2019). The Chinese version of the Montréal Cognitive Assessment Scale-B (MoCA-B) is a reliable cognitive screening test across all education levels in Chinese adults, with high acceptance and good reliability (Yu et al., 2014; Zhou et al., 2014; Chen et al., 2016; Hong et al., 2022). It was administered as neuropsychological screening tests and criteria for grouping in this study. All participants performed a fully standardized cognitive assessment that covered various cognitive domains. General cognitive function was assessed by the Chinese version of the MoCA-B. The total scores range from 0 to 30 points, where higher scores indicate better cognitive function. Participants were grouped into the Dementia (DM), mild cognitive impairment (MCI) group, and normal cognitive state(N) group, with corresponding MoCA scores of ≤18, 19–25, and ≥26, respectively, according to the MoCA scores (Yu et al., 2014; Chen et al., 2016).

The Rey Auditory Verbal Learning test (RAVLT) and clinical dementia rating (CDR) scale were also administered as neuropsychological screening tests in this study (Molloy et al., 1991; Rai, 1993; Li et al., 2016; Arevalo-Rodriguez et al., 2021). Verbal memory was evaluated with the RAVLT, including the total score and the short delay recall, Wired quiz -A and B, digit span task, clock draw test, Long-delayed recall and cue recall (after an interval of 20 min).

The CDR scale is widely used in clinical trials for staging the severity of AD and other dementias (Lanctôt et al., 2009; Wessels et al., 2018). It is comprised of six cognitive domains, including memory, orientation, ability to judge and solve problems, community affairs, housework and hobbies, and personal care ability. After interviewing both participants and their informants, trained physicians scored their points. Each domain is rated on a 5-point scale independently from each others, except the personal care domain which is a 4-point scale without the 0.5 rating scale. Point 0 represents no impairment, 0.5 represents questionable/very mild impairment, 1 represents mild impairment, 2 represents moderate impairment, and 3 represents severe impairment. The diagnosis of MCI or dementia can be confirmed after the assessment (Albert et al., 2011; McKhann et al., 2011).

### 2.4 Image acquisition

A 3T scanner (Philips Achieva 3.0T, Philips Medical Systems, Best, Netherlands) with an 8-channel head coil was employed to acquire each participant’s whole brain MRI data. Axial Fluid Attenuated Inversion Recovery (FLAIR) was used as an MRI study sequence. FLAIR images were acquired using FLAIR_LongTR sequences (TR = 9000 ms, TE = 120 ms, flip angle = 90°, Prep Time = 450 ms, slice thickness = 2mm, slice gap = 0 mm, number of excitations (NEX) = 1, field of view (FOV) = 256 mm × 256 mm, matrix size = 256 × 238, axial slices = 100). To suppress head motion artifacts, foam pads were used to fix the head during scanning. The participants lie in a supine position, keep their eyes closed and awake, and try to avoid ideological activities following the operator’s instructions. In the whole process of scanning, the participants and their images quality were monitored by two experienced radiologists.

If the images were abnormal or the participants were uncomfortable, the acquisition would be terminated immediately. For each participant, we kept the MRI scan on the same day with neuropsychological tests, and within 1 week after the medical history interview, neurological examination, and laboratory examinations.

### 2.5 Image processing

**FIGURE 1:**
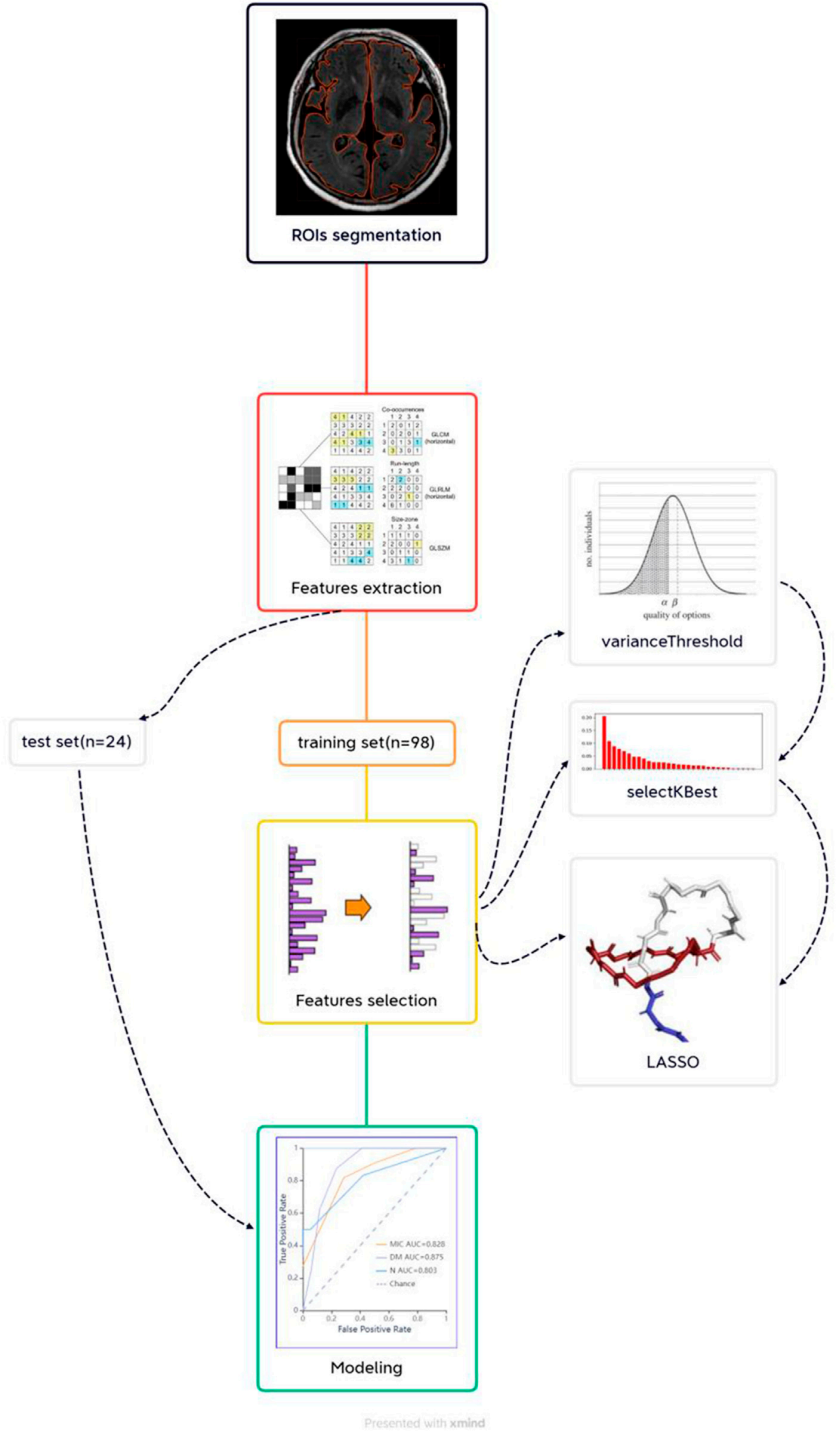
Radiomics workflow.

Figure 1 presents the radiomics workflow, which involves: Imaging acquisition, ROI segmentation, feature extraction and analysis, then developing a statistical model based on ML algorithms (Yamamoto and Hasegawa, 2017; Cheng and Hua, 2020; Mayerhoefer et al., 2020). Radcloud radiomics platform (Huiying Medical Technology, Beijing, China) was employed to analyze the MRI and clinical data, and subsequently to perform radiomics statistics analysis. The original images of the participants, exported from MRI scanners in DICOM format, were uploaded to the Radcloud platform for the next step of the analysis.

#### 2.5.1 Image segmentation

We used all MRI-FLAIR sequences in the brain of the subjects as ROIs, and all these images were reviewed by two senior radiologists with 12 (reader 1) and 9 years (reader 2) experience in radiology. The cerebrum in the FLAIR sequences of the participants was delineated manually and layer-by-layer by the two radiologists who were blinded to their clinical information of them, then all contours were reviewed by a third senior radiologist with 17 years of experience in this field. Thus, the ROIs contained all the components of the cerebrum, without the Cerebellum, brainstem, ventricles, and other unrelated structures. Agreement of the VOIs between the two radiologists who delineated manually the ROIs was evaluated by intraclass correlation coefficient (ICC), ICC >0. 80 indicates good consistency. Eventually, 122 VOIs were segmented from 122 patients’ MRI scans which were used for subject analysis.

#### 2.5.2 Feature extraction

In total, 1,409 radiomics features were extracted from the MRI with the Radcloud platform (https://mics.huiyihuiying.com/). These features were comprised of three groups. Group 1 (first-order statistics) contained 126 descriptors that quantitatively delineate the distribution of voxel intensities within the MR image through commonly used and basic metrics. Group 2 (shape- and size-based features) consisted of 14 three-dimensional features that reflect the shape and size of the region. From grey-level run-length and grey-level co-occurrence texture matrices, 525 textural features that can quantify region heterogeneity differences were classified into group 3 (texture features).

#### 2.5.3 Feature selection

As described above, large amounts of radiomics features were extracted from the MR images of participants. However, not all these features were useful for the construction of the ML model. Therefore, for the best performance of the model, dimensionality reduction for the selection of task-specific features is an essential procedure. The feature selection methods for reducing the redundant features included the variance threshold (variance threshold = 0.8), SelectKBest, and the least absolute shrinkage and selection operator (LASSO) algorithm. For the variance threshold method, the threshold is 0.8, so the features of the variance bigger than 0.2 were used. The SelectKBest method, which belongs to a single variable feature selection method, employs a *p*-value to analyze the relationship between the features and the results of classification, all the eigenvalues with a *p*-value smaller than 0.05 will be retained. For the LASSO model, the L1 regularizer was used as the cost function, the error value of cross-validation is 5, and the maximum number of iterations is 1,000. After the three-step dimensionality reduction, the remaining features with the greatest correlation were used to construct a radiomics model employing a ML algorithm.

#### 2.5.4 Development of MRI-based ML model

In this study, The validation dataset and training dataset were divided by random method with a ratio of 2:8, and the random seeds are 500. Based on the selected optimal features, we used 3 ML classifiers available for classification analysis, which creates radiomics models that attempt to separate the data concerning cognitive function in patients with T2DM. The three classifiers included k-NearestNeighbor (KNN), Support Vector Machine (SVM), and Logistic Regression (LR). The validation method was used to improve the efficiency of these models.

Both The KNN and SVM are a type of supervised learning method. The KNN attempts to predict the correct class for the validation set by calculating the distance between the Validation set and the training set. For KNN, the parameters KNN parameters: n_neighbors (5), weights (uniform) in this study. The SVM tries to search for an optimal separating hyperplane between classes, which maximizes the margin. For SVM, the parameters SVM parameters: kernel (rbf), C (1), gamma (auto), class_weight (balanced), decision_function_shape (ovr) in this study. The LR is a statistical method used to evaluate the correlation between the dependent and independent variables. For LR, the parameters LR parameters: penalty (L2), C (1), solver (liblinear), class_weight (None), multi_class (ovr) in this study.

### 2.6 Statistical analysis

All the clinical data analyses were performed by IBM SPSS Statistic version 22.0 (SPSS Inc.). Comparisons of clinical and demographic features of patients among the three groups were conducted by Chi-squared test, and one-way analysis of variance (ANOVA) followed by LSD test or Tamhane’s T2 test, A significant statistical difference was presented as *p* < 0.05. Partitions of χ^2^ method calibration and inspection level *α*’ = 0.017. Statistical analyses were performed for both the training and validation sets. Dice’s coefficients were used to evaluate the intra- and inter-observer consistency for the ROI segmentation and radiomics feature extraction with 30 randomly selected samples. We interpreted a coefficient of 0.81–1.00 was interpreted as perfect agreement.

The statistical analysis of ML models was performed in the Radcloud platform. The receiver operating characteristic (ROC) curve which presented the area under the curve (AUC), was used both in the training and validation set respectively to evaluate the predictive performance. To evaluate the performance of classifier, four indicators including P (precision = true positives/(true positives + false positives)), R (recall = true positives/(true positives + false negatives)), f1-score (f1-score = P*R*2/(P + R)), support (total number in test set) were used in this study. The precision is the overall evaluation of the classifier and represents the proportion of correctly divided samples to the determined divided samples. The f1-score is used to evaluate the classification efficiency of the classifier. The higher the F1 value, the better the classification effect. The AUC evaluates the classifier’s performance. The optimal classifier was determined by the principle of maximizing the Youden index (ie, sensitivity + specificity-1).

## 3 Results

### 3.1 Characteristics of the study population

In this study, A total of 122 participants were analyzed: 40 participants in the DM group, 52 in the MCI group, and 30 in the N group. The mean age of the subjects was 63 (7.07) years, 47 men and 75 women.

Table 1 summarizes the clinical and demographic characteristics of the MCI, DM, and N groups. No significant differences were found in BMI, systolic blood pressure, FBG, ALT, AST, GGT, BUN, BUA, SCr, TG, TC, LDL, HDL, UCr, Coronary heart disease, history of smoking, retinopathy, Intermittent claudication and duration of diabetes (*p* > 0.05). Compared with the N group, the DM group had a higher level of age (*p* = 0.013), Diastolic blood pressure (*p* = 0.019), HbA1c (*p* = 0.001), and lower level of gender (male) (*p* = 0.003). Compared with the N group, both the DM and MCI group had lower levels of MoCA, RAVLT (*p* < 0.001, *p* < 0.001 and *p* < 0.001, *p* < 0.001, respectively). Compared with the MCI group, the DM group also had lower levels of MoCA (*p* < 0.001) and RAVLT (*p* = 0.003). Compared with the DM group, both the MCI and N groups had lower levels of CDR (*p* = 0.002, *p* = 0.005, respectively). Furthermore, compared with the N group, the MCI group had a higher level of HbA1c (*p* = 0.020).

**TABLE 1:** Demographic and clinical characteristics of the MCI, DM, and N groups.

Characteristics	DM (*n* = 40)	MCI (*n* = 52)	N (*n* = 30)	F/χ^2^	*p*-value
Age (years)^d^	65.08 ± 7.38^a^	62.67 ± 6.90	60.77 ± 6.83	3.30	0.04
Gender (male)^e^	8 (11.1%)^a^	16 (18.2%)	15 (33.3%)	8.95	0.01
MoCA (scores)^d^	14.83 ± 2.90^ab^	22.06 ± 2.25^c^	26.73 ± 0.98	276.37	0.00
RAVLT (scores)^d^	10.03 ± 3.839^ab^	13.12 ± 5.29^c^	18.20 ± 4.92	25.31	0.00
ADLs (scores)^d^	21.92 ± 6.310	20.09 ± 0.570	20.03 ± 0.183	2.21	0.14
CDR (scores)^d^	0.31 ± 0.74^ab^	0.03 ± 0.12	0.02 ± 0.091	5.85	0.01
BMI (kg/m2)^d^	27.38 ± 3.36	26.89 ± 3.08	27.03 ± 2.37	0.30	0.74
Systolic blood pressure (mmHg)^d^	121.23 ± 16.19	124.94 ± 13.54	126.87 ± 13.10	1.45	0.24
Diastolic blood pressure (mmHg)^d^	65.75 ± 13.26^a^	70.35 ± 9.16	72.1 ± 10.85	3.25	0.04
FBG (mmol/L)^d^	9.29 ± 3.08	8.95 ± 2.90	8.26 ± 2.31	1.16	0.32
HbA1c (mmol/mol)%^d^	8.088 ± 1.69^a^	7.71 ± 1.33^c^	6.94 ± 1.21	5.65	0.01
ALT (U/L)^d^	31.83 ± 21.13	30.04 ± 17.61	27.11 ± 12.80	0.60	0.55
AST (U/L)^d^	24.68 ± 11.21	23.64 ± 11.62	21.45 ± 6.58	0.83	0.44
GGT (U/L)^d^	44.25 ± 47.76	48.04 ± 81.28	37.87 ± 37.20	0.25	0.78
BUN (mmol/L)^d^	6.32 ± 6.81	5.18 ± 1.44	4.74 ± 1.12	1.46	0.24
SCr (μmol/L)^d^	62.90 ± 13.61	67.65 ± 16.10	66.10 ± 12.85	1.15	0.32
TG (mmol/L)^d^	2.26 ± 1.74	2.03 ± 1.23	2.77 ± 2.33	1.50	0.23
TC (mmol/L)^d^	4.34 ± 1.22	4.13 ± 0.99	4.59 ± 1.30	1.51	0.23
LDL (mmol/L)^d^	2.27 ± 0.98	2.30 ± 0.82	2.59 ± 0.96	1.23	0.30
HDL (mmol/L)^d^	1.14 ± 0.29	1.06 ± 0.28	1.08 ± 0.22	1.01	0.37
UCr (mmol/L)^d^	5.79 ± 3.50	7.89 ± 7.07	6.66 ± 9.80	3.01	0.14
duration of diabetes (years)^d^	15.86 ± 9.59	21.61 ± 19.37	18.23 ± 2.69	0.298	0.74
Coronary heart disease^e^	14 (19.4%)	22 (25.0%)	12 (26.7%)	1.02	0.60
Smoker,now^e^	5 (6.9%)	14 (15.9%)	6 (13.3%)	3.04	0.22
Smoker, past^e^	8 (11.1%)	20 (22.7%)	12 (26.7%)	5.28	0.07
Retinopathy^e^	2 (2.8%)	1 (1.1%)	3 (6.7%)	3.21	0.20
Intermittent claudication^e^	5 (6.9%)	4 (4.5%)	0 (0%)	3.19	0.20

^d^ Data are presented as mean ± SD.

^e^ Data are presented as cases (percentage%).

a, b, and c represents a statistically significant difference between the DM and N group, the DM group and MIC, as well as the MIC and N group, respectively. The test level of gender was α, < 0.017. *p* < 0.05 was considered significant. DM, dementia; MIC, mild cognitive dysfunction; N, normal cognitive state; MoCA, montreal cognitive assessment scale, RAVLT, rey auditory verbal learning test; ADLs, Activities of Daily Living; CDR, clinical dementia rating scale; FBG, fasting blood-glucose; HBALC, glycated hemoglobin; GGT, gamma-glutamyltransferase; AST, glutamic oxalacetic transaminase; BUN, blood urea nitrogen; BUA, blood uric acid; TC, total cholesterol; SCr, serum creatinine; UCr, Urine creatinine.

### 3.2 MRI-based radiomics features

The inter-observer and intra-observer agreement was perfect for the segmentation of the VOIs for the MRI-FLAIR images. The VOIs had a better correlation between the two radiologists (ICC: 0.897; 95% CI: 0.829–0.932).

**FIGURE 2:**
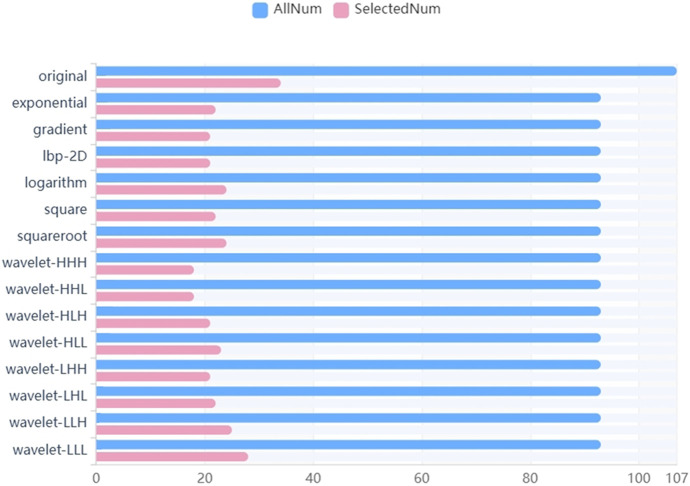
Variance threshold on feature selection. We used variance threshold methods to select radiomics features (variance threshold = 0.8), and we selected 344 features from 1,409 features.

**FIGURE 3:**
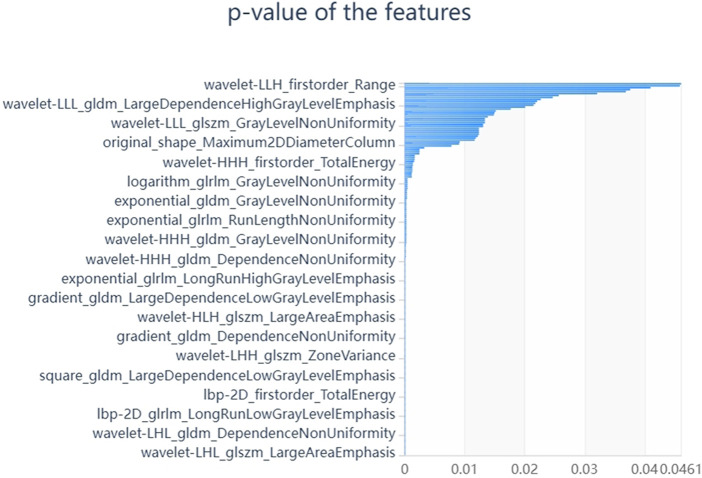
Select K best on feature selected. We used Select K best methods to further select radiomics features, we selected 192 features.

**FIGURE 4:**
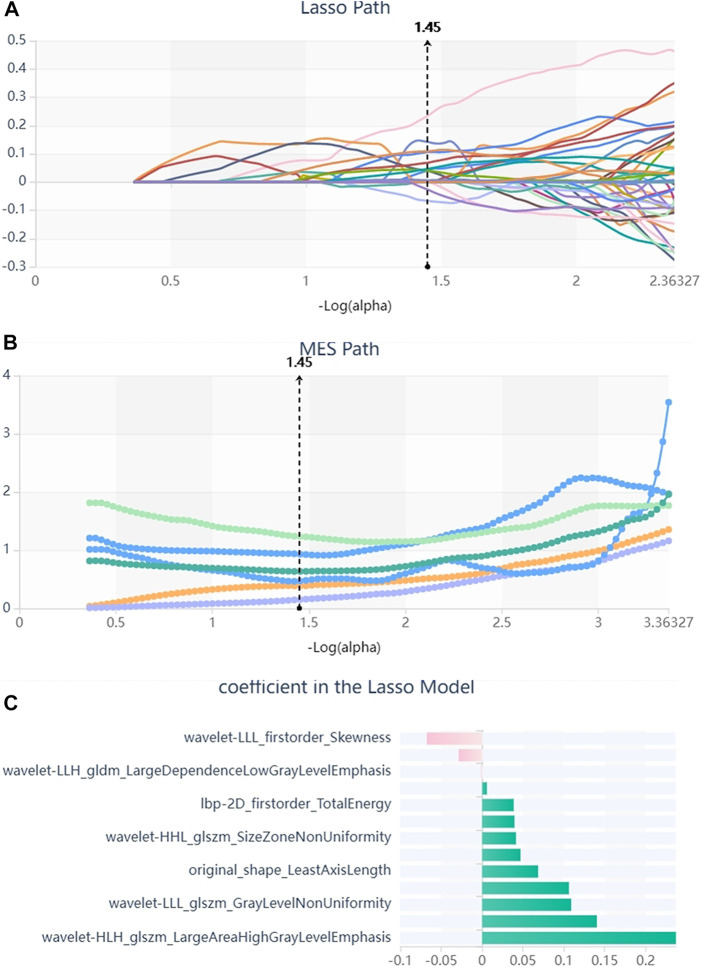
Lasso algorithm on feature selected. **(A)** Lasso path; **(B)** MSE path; **(C)** coefficients in Lass model. Using the Lasso model, 13 features that correspond to the optimal alpha value were selected.

We firstly selected 344 features from 1,409 features using the variance threshold method (Figure 2), then with the Select-K best methods, we selected 192 features (Figure 3), and finally, 13 most relevant features were selected with the LASSO algorithm (Figure 4). Details of the selected 18 features were shown in Table 2.

**TABLE 2:** Description of the selected radiomics features with their associated feature group and filter.

Radiomic feature	Radiomic class	Filter
LargeAreaHighGrayLevelEmphasis	glszm	wavelet-HLH
SizeZoneNonUniformity	glszm	wavelet-HHL
LargeAreaEmphasis	glszm	wavelet-LHL
TotalEnergy	firstorder	lbp-2D
SizeZoneNonUniformity	glszm	wavelet-HLH
LeastAxisLength	shape	original
Skewness	firstorder	wavelet-LLL
GrayLevelNonUniformity	glszm	wavelet-LLL
Kurtosis	firstorder	wavelet-LLH
LargeDependenceLowGrayLevelEmphasis	gldm	wavelet-LLH
Maximum	firstorder	wavelet-LLH
Range	firstorder	wavelet-LLH
Energy	firstorder	wavelet-HLL

### 3.3 MRI-based ML model evaluation and comparison

All the participants were divided into training (*n* = 98) and validation (*n* = 24) sets. Three ML algorithms, including the KNN, SVM, and LR, were applied for the construction of the prediction models in the training set. To evaluate the prediction model in test data, the ROC curve, sensitivity, specificity, Precision, F1-score, and Support were used. The analysis results of the ROC curve were displayed in Table 3 for the training set and Table 4 for the validation set. Before the assessment, the Youden index by the ROC curve in the validation set was employed to determine the optimal algorithm. The KNN model was better than those of other classifiers, with Youden index of 0.59 in DM, 0.57 in MCI, and 0.62 in N respectively.

**TABLE 3:** ROC results with KNN, SVM, and LR classifiers of the training set.

Classifiers	Category	AUC	95% CI	Sensitivity	Specificity	Youden index
KNN	DM	0.928	0.830–0.932	0.780	0.830	0.61
	MCI	0.837	0.745–0.929	0.710	0.750	0.46
	N	0.924	0.815–0.941	0.670	0.970	0.64
SVM	DM	0.974	0.888–0.982	0.840	0.920	0.76
	MCI	0.939	0.868–0.984	0.850	0.820	0.67
	N	0.983	0.882–0.993	0.750	0.970	0.72
LR	DM	0.979	0.893–0.995	0.840	0.970	0.81
	MCI	0.920	0.856–0.984	0.930	0.800	0.73
	N	0.926	0.820–0.978	0.750	0.990	0.74

**TABLE 4:** The results of AUC, 95% CI, Sensitivity, and Specificity in the validation cohort.

Classifiers	Category	AUC	95% CI	Sensitivity	Specificity	Youden index
KNN	DM	0.875	0.678–0.948	0.750	0.880	0.63
	MCI	0.828	0.655–0.962	0.820	0.710	0.53
	N	0.803	0.569–0.921	0.500	0.950	0.45
SVM	DM	0.846	0.674–0.984	0.880	0.760	0.64
	MCI	0.857	0.687–0.947	0.730	0.860	0.59
	N	0.825	0.625–0.938	0.500	0.950	0.45
LR	DM	0.831	0.662–0.974	0.880	0.710	0.59
	MCI	0.883	0.719–0.948	0.640	0.930	0.57
	N	0.904	0.791–0.991	0.670	0.950	0.62

**FIGURE 5:**
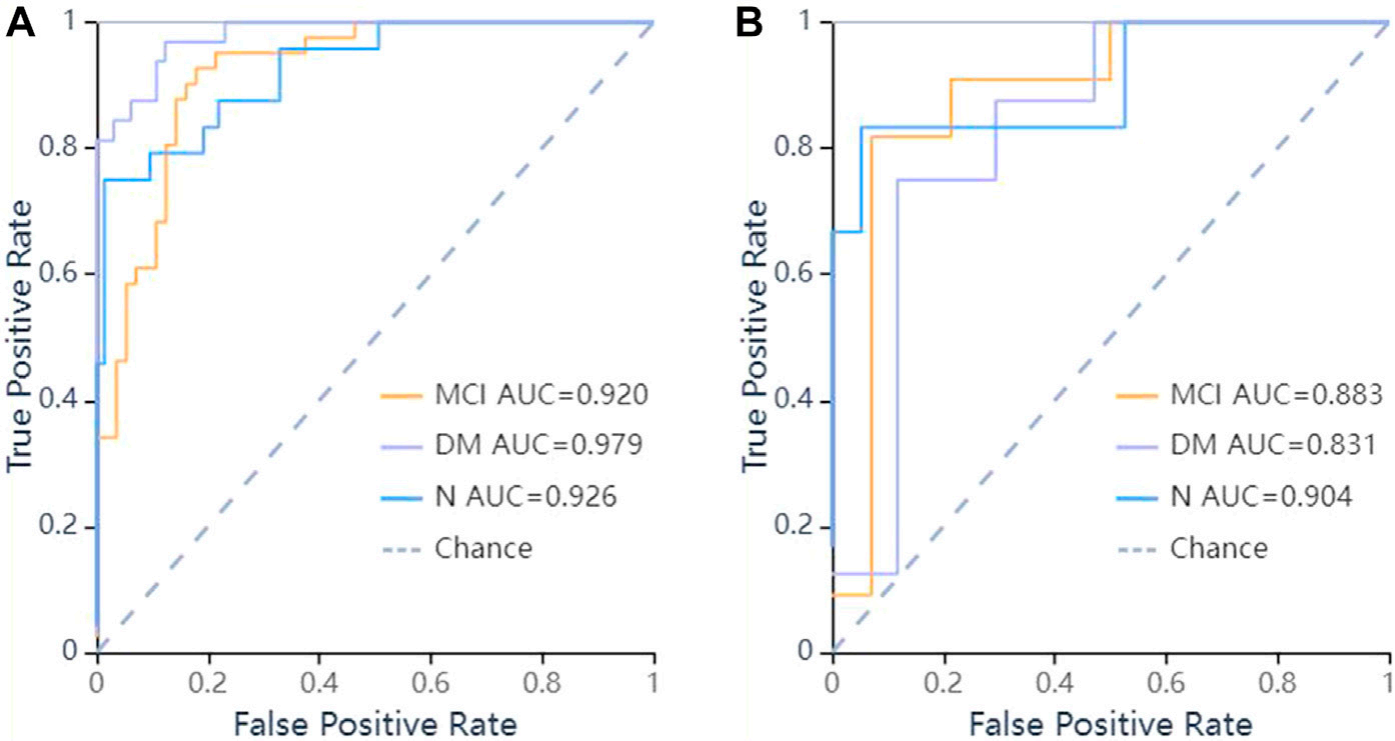
ROC curves of LR methods to classification. **(A)** ROC curve of training set, the AUC were 0.979 in DM (sensitivity and specificity were 0.84 and {"MCI”: 0.8, “DM”: 0.97, “N": 0.99}), 0.92 in MCI (sensitivity and specificity were 0.93 and {"MCI”: 0.8, “DM”: 0.97, “N": 0.99}), 0.926 in N (sensitivity and specificity were 0.75 and {"MCI”: 0.8, “DM”: 0.97, “N": 0.99}) respectively; **(B)** ROC curve of validation set, the AUC were 0.831 in DM (sensitivity and specificity were 0.88 and {"MCI”: 0.93, “DM”: 0.71, “N": 0.95}), 0.883 in MCI (sensitivity and specificity were 0.64 and {"MCI”: 0.93, “DM”: 0.71, “N": 0.95}), 0.904 in N (sensitivity and specificity were 0.67 and {"MCI”: 0.93, “DM”: 0.71, “N": 0.95}) respectively.

**FIGURE 6:**
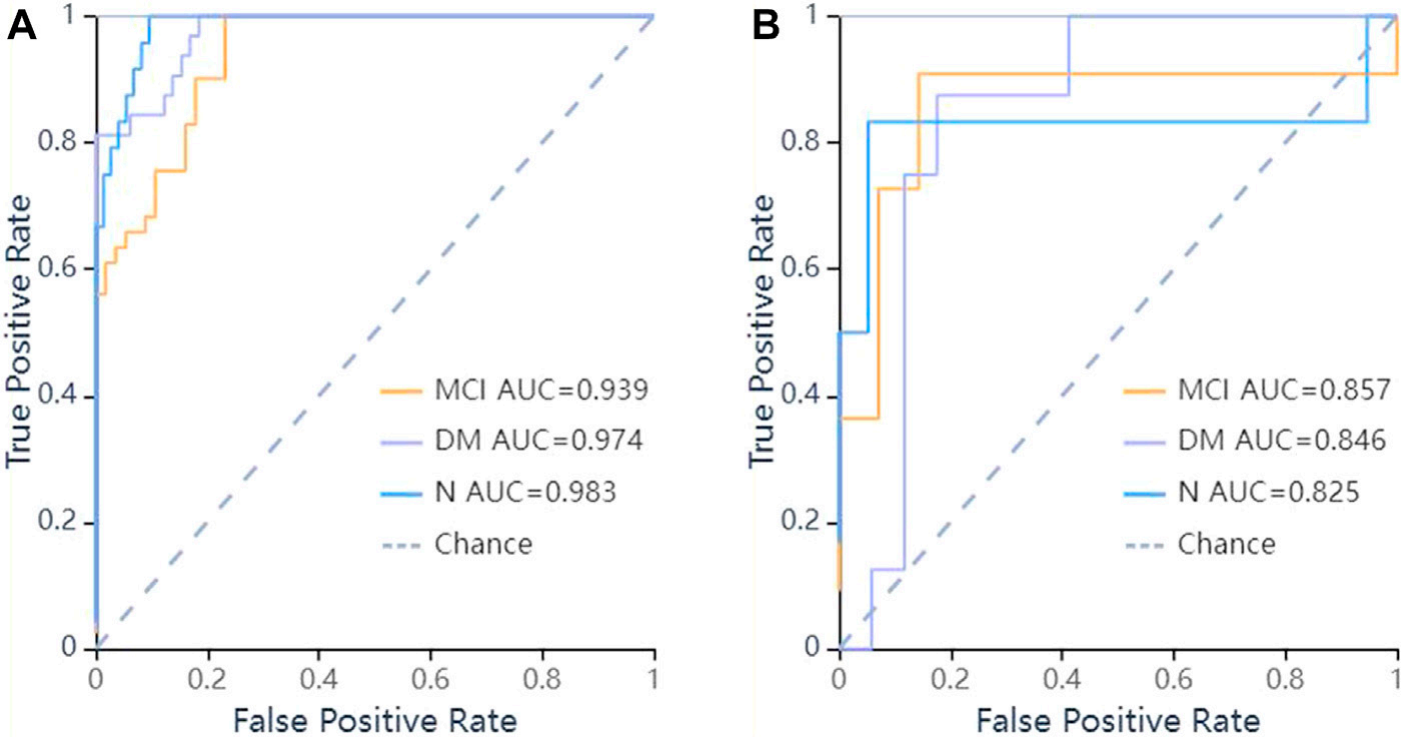
ROC curves of SVM methods to classification. **(A)** ROC curve of training set, the AUC were 0.974 in DM (sensitivity and specificity were 0.84 and {"MCI”: 0.82, “DM”: 0.92, “N": 0.97}), 0.939 in MCI (sensitivity and specificity were 0.85 and {"MCI”: 0.82, “DM”: 0.92, “N": 0.97}), 0.983 in N (sensitivity and specificity were 0.75 and {"MCI”: 0.82, “DM”: 0.92, “N": 0.97}) respectively; **(B)** ROC curve of validation set, the AUC were 0.846 in DM (sensitivity and specificity were 0.88 and {"MCI”: 0.86, “DM”: 0.76, “N": 0.95}), 0.857 in MCI (sensitivity and specificity were 0.73 and {"MCI”: 0.86, “DM”: 0.76, “N": 0.95}), 0.825 in N (sensitivity and specificity were 0.50 and {"MCI”: 0.86, “DM”: 0.76, “N": 0.95}) respectively.

**FIGURE 7:**
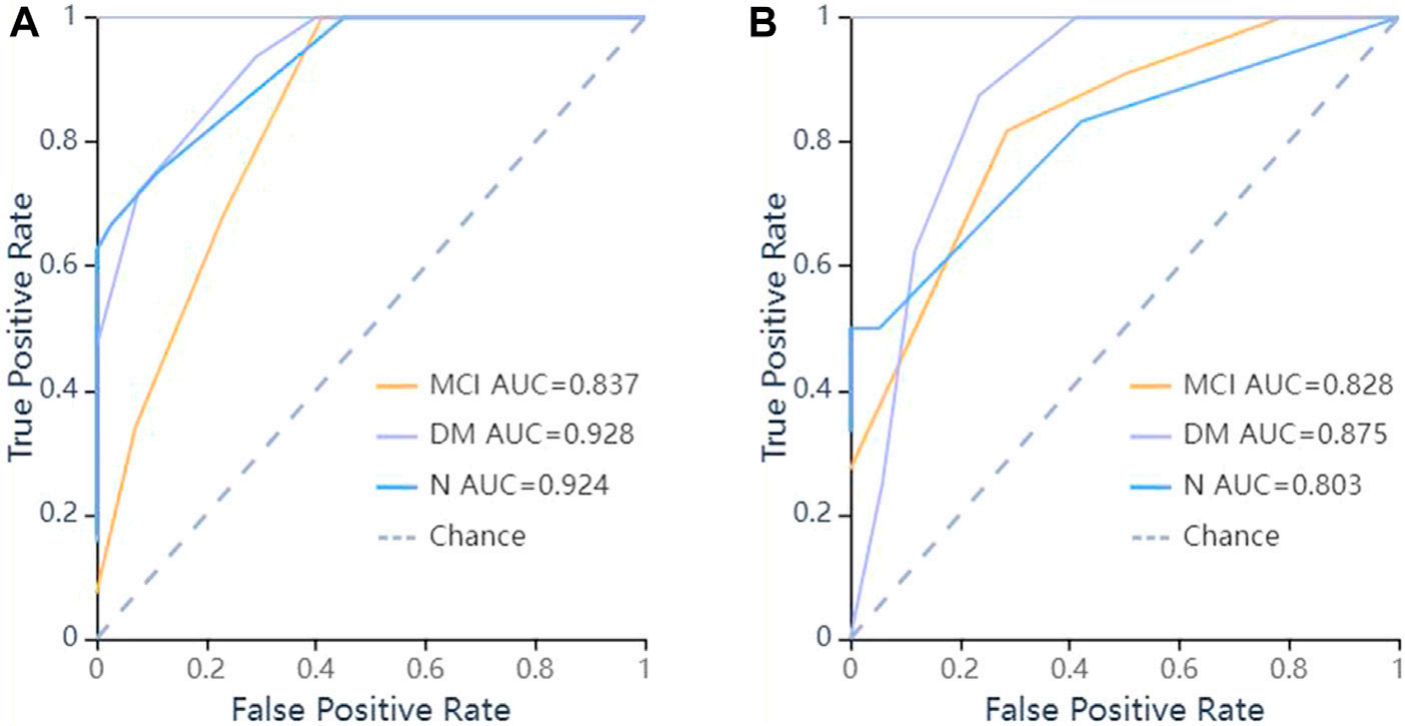
ROC curves of KNN methods to classification. **(A)** ROC curve of training set, the AUC were 0.928 in DM (sensitivity and specificity were 0.78 and {"MCI”: 0.75, “DM”: 0.83, “N": 0.97}), 0.837 in MCI (sensitivity and specificity were 0.71 and {"MCI”: 0.75, “DM”: 0.83, “N": 0.97}), 0.924 in N (sensitivity and specificity were 0.67 and {"MCI”: 0.75, “DM”: 0.83, “N": 0.97}) respectively; **(B)** ROC curve of validation set, the AUC were 0.875 in DM (sensitivity and specificity were 0.75 and {"MCI”: 0.71, “DM”: 0.88, “N": 0.95}), 0.828 in MCI (sensitivity and specificity were 0.82 and {"MCI”: 0.71, “DM”: 0.88, “N": 0.95}), 0.803 in N (sensitivity and specificity were 0.50 and {"MCI”: 0.71, “DM”: 0.88, “N": 0.95}) respectively.

In the validation set, the ROC curves revealed that the LR algorithm had the best predictive performances, with an area under the curve (AUC) of 0.831 in DM, 0.883 in MCI, and 0.904 in the normal cognitive group(N). Relatively, the SVM and KNN algorithms had the second and third predictive performances respectively. In the SVM model, the AUCs of the test set were 0.846 in DM, 0.857 in MCI, 0.825 in N respectively, and in the KNN model, they were 0.875 in DM, 0.828 in MCI, 0.803 in N respectively (Figures 5–7).

Table 5 summarized these four indicators (precision, recall, f1-score, support) for the three classifiers. When training with LR classifier, the precision, recall, f1-score and support of training set were 0.93, 0.84, 0.89 and 32 in DM, 0.78, 0.93, 0.84 and 41 in MIC, 0.95, 0.75, 0.84 and 24 in N, the precision, recall, f1-score and support of validation set were 0.58, 0.88, 0.70 and 8 in DM and 0.88, 0.64, 0.74 and 11 in MIC and 0.80, 0.67, 0.73 and 6 in N. The results unveiled that the models could differentiate cognitive dysfunction from normal cognitive state and roughly assess its severity in patients with T2DM, and the LR model outperformed other models.

**TABLE 5:** The results of four indicators -Precision, Sensitivity, F1-score, Support in training, and validation set.

	Indicators	Training set			Validation set		
		KNN	SVM	LR	KNN	SVM	LR
DM	Precision	0.690	0.840	0.930	0.750	0.640	0.580
	Sensitivity	0.780	0.840	0.840	0.750	0.880	0.880
	F1-score	0.740	0.840	0.890	0.750	0.740	0.700
	Support	32	32	32	8	8	8
MCI	Precision	0.670	0.780	0.780	0.690	0.800	0.880
	Sensitivity	0.710	0.850	0.930	0.820	0.730	0.640
	F1-score	0.690	0.810	0.840	0.750	0.760	0.740
	Support	41	41	41	11	11	11
N	Precision	0.890	0.900	0.950	0.750	0.750	0.800
	Sensitivity	0.670	0.750	0.750	0.500	0.500	0.670
	F1-score	0.760	0.820	0.840	0.600	0.600	0.730
	Support	24	24	24	24	24	24

## 4 Discussion

In this study, MRI-based ML models were constructed to screen out MCI and dementia in patients with T2DM, which was always performed by neuropsychological screening tests in clinical practice. Our research results demonstrated that ML models, which can extract high dimensional radiomics features from conventional FLAIR sequences of MRI, were able to distinguish dementia and MCI from the normal cognitive state in patients with T2DM. LR outperformed other classifiers with the highest predictive performances, and its AUCs were 0.881 for MCI, 0.883 for dementia, and 0.904 for the normal cognitive state, respectively.

This study shows that several clinical characteristics may be risk factors for cognitive dysfunction in patients with T2DM. Cognitive impairment in patients with T2DM is increasingly recognized and taken seriously. Moreover, with aging, the incidences of both T2DM and dementia increase, which contributes to the prevalence of the comorbidity of these pathologies. A meta-analysis confirmed that the incidence of MCI in patients with T2DM was approximately 45.0% (95% CI = 36.0, 54.0) (You et al., 2021). Multiple pieces of evidence have indicated that T2DM is related to vascular dementia (VD) and Alzheimer’s disease (AD) (Chau et al., 2020; Lyu et al., 2020; Ortiz et al., 2022). Recent studies have revealed that older patients with T2DM have a higher risk of MCI or dementia, compared to young patients with a similar condition (Sanke et al., 2014; Gao et al., 2016; Machii et al., 2020; Suain Bon et al., 2021). The result was consistent with our study which demonstrated that there is a significant difference between the N and DM groups. In other words, aging may be a risk factor for cognitive dysfunction in patients with T2DM. In addition, the duration of diabetes may be related to cognitive dysfunction. Previous studies have shown that there is a linear association between the duration of T2DM and the decline in cognition (Tuligenga et al., 2014; Wood et al., 2016). However, our study did not demonstrate significant differences among the MCI, dementia, and normal cognitive state in patients with T2DM (*p* < 0.05), which may owe to the small sample size and selection bias and needs to be further confirmed by some more advanced methods. Moreover, this study demonstrated that HbA1c was associated with an increased risk of MCI and dementia. Yaffe K et al. studied 1983 postmenopausal women with osteoporosis who had HbA1C levels measured at baseline. Development of mild cognitive impairment (MCI) or dementia over 4 years was determined as part of a dementia ancillary study. They found an association between HbA1C level and the risk of developing MCI or dementia in postmenopausal osteoporotic women primarily without diabetes (Yaffe et al., 2006).

In clinical practice, various strategies such as neuropsychological screening tests, medical history, and brain imaging findings, have been proposed to identify cognitive impairment in patients. Recently, some studies suggested that both Amyloid-βand tau has helped assess cognitive dysfunction, but the data for such biomarkers are not easy to be acquired clinically (Groot et al., 2021). Neuropsychological screening tests are easily performed in most clinics or hospitals, but the fact that the neuropsychological data were difficult to be interpreted has increased the necessity of medical images, artificial intelligence, and even a combination of them. In the field of medical image, several Cognitive dysfunction biomarkers have been studied including the brain metabolic change derived from fluorodeoxyglucose positron emission tomography (FDG-PET) (Yuan et al., 2009; Pardo et al., 2010; Newberg et al., 2022), and the structural or functional change in the brain measured by MRI (van Harten et al., 2007; Belfort-Deaguiar et al., 2014; Lei et al., 2021). In recent years, with the development of MRI scanner and its increasingly enhanced data processing function, the level of analysis has been moving from assessment of brain structure and morphology such as volume and general atrophy to more detailed and in-depth analysis of white matter tracts, using diffusion tensor imaging (DTI), a newer method. These neuroimaging studies had the potential to identify both functional and structural brain abnormalities that may serve as early biomarkers for cognitive dysfunction.

The mechanisms of cognitive impairment in diabetic patients need to be further studied since it is still unclear so far, and radiomics combined with ML may be useful. For cognition in patients with T2DM, previous research based on DTI revealed that abnormalities in brain structural and functional connectivity are related to widely cognitive impairments (McCrimmon et al., 2012; Biessels, 2013; Biessels and Reijmer, 2014; Yang et al., 2020). Diabetes may be a risk factor for white matter (WM) disease (Ogama et al., 2018; Werhane et al., 2021). Multiple neuroimaging studies have revealed that abnormalities in the WM tract are related to the dysfunction of glucose metabolism and cognition. (van Bussel et al., 2016). A series of disorders with cognitive impairment, including T2DM, has previously been observed to be associated with impaired connectivity of the default mode network (DMN), (Chen et al., 2019; Zarifkar et al., 2021; Magalhães et al., 2022). The structural and DMN connectivity abnormalities observed in DM(Yin et al., 2016; Crockett et al., 2017; McKinnon et al., 2017), but further investigation of the mechanisms of DMN impairment is needed. Resting-state functional magnetic resonance imaging (rs-fMRI) technique can be used to explore the topological properties of functional whole-brain networks. Qin et al. (2019) revealed the abnormalities of the topological properties of whole-brain networks in T2DM patients with theoretical graph analysis using an rs-fMRI technique. At the macroscopic level, the brain can be viewed as a network composed of anatomically separated brain regions, between which the information is transmitted based on the white matter.

The hippocampus has been confirmed long ago as one of the most important brain regions associated with cognition. In patients with T2DM, it was also related to cognitive impairment, and the disruptions of structural and functional connectivity are identified in it (Wang et al., 2014; van Bussel et al., 2016). Sun et al. revealed that the reduced functional connectivity of the hippocampus may be closely related to the disruption of white matter integrity (Sun et al., 2018). Wang et al. combined the textural features and structural images in the hippocampus to investigate their diagnostic performance for AD and MCI using multimodal radiomics technique, and found that the textural features reflecting local functional activity could improve the diagnostic performance of traditional structural models for both AD and MCI (Wang et al., 2022). This study may lay the groundwork for future research on the brain structural and functional connectivity by radiomics methods since the abnormalities in the brain of patients with T2DM were identified by both the DTI and ML model for the whole cerebrum.

Compared to the MRI-based investigations above on the evaluation of cognitive performance in patients with T2DM, our study had an improvement. We utilized the MRI-based ML model as a direct predictor of cognitive performance in patients with T2DM. In the process of research, the selection of features or variables is of great importance in the construction of a prediction model, which can successfully interpret data, with improved classification performance (Saeys et al., 2007; Handelman et al., 2018; Rajkomar et al., 2019). The data employed in our study were acquired from MRI-FLAIR sequence using radiomics features-extracted software, the Radcloud. In the process of feature extraction, a total of 1,409 features were extracted from the original images in DICOM format. In the step of selecting radiomics features, to reduce redundant features for avoiding the curse of dimensionality, we successively used three feature dimension reduction methods, including the variance threshold (variance threshold = 0.8), SelectKBest, and LASSO. Eventually, 13 optimal descriptive radiomics features were enrolled in the model that showed promising predictive performance for the assessment of cognitive function in patients with T2DM, as mentioned above. These radiomics features reflected intrinsic information as textural features that cannot otherwise be detected by radiologists (Loizou et al., 2020; Ta et al., 2020). The first-order statistical features reflect the internal voxel intensity ofthe lesions, and the texture features reflect the gray distribution characteristics in dimensional space, suggesting the heterogeneity of the lesions. Among the 13 features, 6 first-order features, 6 texture features, and 1 shape features comprised the optimal feature set, indicating different feature dimensions to be considered among the DM, MCI, and normal cognitive state in patients with T2DM.

In model establishment, we trained three classifiers, including the KNN, LR, and SVM. LR had the best prediction performance among the three classifiers. Because radiomics contain multiple high-dimensional data, proper strategies for feature selection and model classifiers are required, and machine learning algorithms can be effective for these purposes. LR is a traditional statistical method by obtaining interpretable estimates of the nature and statistical significance of associations between predictors and the outcome. LR is an excellent machine learning algorithm because it is a statistical method used to evaluate the correlation between the dependent and independent variables. Both LR and SVM are linear classification algorithms if the kernel function is not considered. However, SVM only considers the points near the local boundary line, while LR considers all. Nevertheless, the difference in performance across algorithms was sometimes small, including when compared to logistic regression. It is not possible to provide precise rules about sample size requirements for supervised learning. In general, prediction performance improves as sample size increases (Shatte et al., 2019; Jiang et al., 2020). In this study, the performance of the MRI-based ML prediction model for cognitive dysfunction such as MCI and dementia in patients with T2DM was explicitly evaluated. As a type of MRI biomarker, the MRI-based ML prediction model is arguably easier to obtain and implement, less expensive, and explains a significant proportion of variation in cognitive performance, compared to the demographic and genetic risk factor data (Ford et al., 2019; Hall et al., 2019).

We acknowledge that there are several limitations to this study. First of all, because it is a retrospective study, the reproducibility and comparability of the results may exist on account of potential selection bias. Thus, further studies may be needed to improve the clinical usefulness of this machine-learning model. Secondly, multicenter studies with a larger sample for further validation of its reproducibility are required in that this study was a single-center experience limited to our institute. Thirdly, manual ROI segmentation is complicated and time-consuming, especially for the connection of the cerebrum and cerebellum without a well-defined boundary, the automatic segmentation technique with satisfactory reliability and reproducibility is needed. In addition, we only used FLAIR sequence, and other sequences such as T1 and T2 weighted images may also contain useful information. In further research, we will explore the useful information of these sequences.

## 5 Conclusion

In conclusion, by MRI-based radiomics features, we constructed a radiomics model to predict cognitive dysfunction in patients with T2DM, and it was shown to have a good performance and may serve as a potential tool to guide personalized treatment. Compared with the SVM and KNN, the LR algorithm for the construction of the model showed better performance. However, more studies with independent replication datasets are needed to confirm our findings, so that the hypothetical prediction model can be used as a clinical tool to screen for cognitive function. It is believed that as an important part of precision medicine, radiomics will be widely used in the diagnosis and evaluation of cognitive states in patients with T2DM in the future.

## Data Availability

The raw data supporting the conclusion of this article will be made available by the authors, without undue reservation.
